# The Period of Incubation of Syphilis

**Published:** 1919-01

**Authors:** 


***The Period of Incubation of Syphilis.*** By A. JAMBON and A. TZANCK. *Paris Médical*, Oct. 26, 1918.

Concerning syphilis, the first period of incubation is considered to exist until the appearance of chancre, then the second period begins with the onset of the roseola.

This incubation in stages makes of syphilis a very special disease. In most infectious diseases the initial lesion remains unknown; the evolution taking place either in the depth of an organ or in the thick tissues of mucus, and the lesion apparently latent clinically, until the first definite symptoms appear.

The ancients used to consider these delays as the proper character of the disease, although they recognized in them various phases of its development.

Such a conception is strengthened: (1) by the modern researches on the humoral modifications in infectious diseases; (2) by the assimilation with the latter of reactions caused by hetero-albuminous injections.

A number of studies, such as those of Metchnikoff, Richet, Ehrlich and others, have altered the established conception as to the humoral pathology of syphilis.

Antibodies are in excess in the blood and it is a fact, not a hypothesis, that there is definite change in the humors of the organism during the " periode d'état ".

In diseases with a rapid onset the appearance of humoral phenomena marks the invasion phase, and fever emphasizes the perturbation of the various regulating automatisms.

In diseases with slow onset, the invasion may pass unseen until a more detached or obvious symptom is noticed, such as the appearance of chancre in syphilis.

According to the old conception initial sclerosis is really the first symptom, but, from the standpoint of general pathology, such is not the case.

Among humoral modifications to be noticed are: (1) In the blood, appearance of the reaction of fixation of the Bordet-Wasserman complement and augmentation of the refractometric power of the serum. (2) In the cephalo-rachidian fluid, modifications of the pressure of the lymphocytosis; of hyperalbuminosis bearing especially on the quantity of globulin; reaction on iodine; stabilizing action on colloidal gold; positive Wasserman in a certain number of cases. All these signs are invariably missing when chancre is first formed. The forty-five days of the second incubation are, therefore, far from being a homogenous period. All the various humoral phenomena come in degrees revealed by such symptoms as cephalic anemia, temperature, roseola. In practice the second period may be said to begin very soon after the 21st, after the appearance of the chancre; that is to say, 40 days after inoculation. Neisser's works give weight to this assertion. It is known that in the presence of a foreign agent the organism can present itself under various states, either receptive or refractory, immunized or sensibilized; namely, constituting what von Pirquet calls " allergia ", and Siebert " anergia ", since the syphilitic is not protected from his own treponema. In the progression of the disease the organism modifies itself, indicating a systematized syphiliplastic reaction.

It is also known that early reinoculations are, in every way, similar to the initial infection, when, on the contrary, late reinoculations present only non-ulcerated tuberculo papulas.

In 6 months time, over 1100 patients have been admitted to the venereal wards. In the out-patient department approximately 25,569 injections were given during that period and the numbers of cases submitted to other treatments varied between 1183 and 11.

One of the most troublesome complications encountered was epididymitis. The measure taken in acute cases was epididymotomy, while in chronic, recurring cases excision was applied. Stricture of the urethra was another frequent complication. The method used consisted in distending the urethra with oil or liquid petroleum and inserting whalebone and rat-tail bougies of increased calibre, which finally worked through the stricture. Sounds were screwed on the rat-tail which guided them into the bladder.

There follows a table of urological and venereal observations.

